# Primary Splenic Angiosarcoma Revealed by Bone Marrow Metastasis

**DOI:** 10.4274/tjh.2013.0049

**Published:** 2014-12-05

**Authors:** Soumaya Anoun, Sofia Marouane, Asmae Quessar, Said Benchekroun

**Affiliations:** 1 Hopital 20 AOUT, Clinic of Hematology and Pediatric Oncology, Casablanca, Morocco; 2 Hopital Ibn Rochd, Clinic of Histopathology, Casablanca, Morocco

**Keywords:** Angiosarcoma, Splenomegaly, Bone marrow infiltration

## Abstract

Primary splenic angiosarcomas are the most common malignant non-hematopoietic tumors of the spleen. Metastatic diseases were found in 69% of patients in a reported series but the incidence of bone marrow involvement is unclear. We report a rare case of a 25-years-old Moroccan woman with unsuspected primary splenic angiosarcoma revealed by bone marrow metastasis. She presented with serious anemia and splenomegaly. Bone marrow biopsy revealed proliferating spindle cells. Computed tomography scanning showed an enlarged spleen with heterogeneous lesions. Splenectomy was performed and retrospective histological study of the spleen confirmed the diagnosis. She died 1 year after splenectomy.

## Abstract (tr)

Primer splenik anjiosarkom dalağın hematolojik olmayan tümörlerinden en sık karşılaşılanıdır. Yayınlanmış serilerde metastaz %69 olarak belirtilmişse de kemik iliği metastazının sıklığı tam bilinmemektedir. Biz Faslı bir kadın hastada kemik iliği metastazı ile açığa çıkan daha önceden şüphelenilmemiş bir primer splenik anjiosarkom vakasını rapor etmek istiyoruz. Yirmi beş yaşındaki hastanın ciddi anemi ve splenomegalisi mevcuttu. Kemik iliği biyopsisinde kemik iliği alanlarının yerini prolifere olmuş iğsi hücrelere bıraktığı gözlendi. Bilgisayarlı Tomografide büyümüş dalak içinde heterojen lezyonlar görüldü. Splenektomi yapıldı ve dalak üzerinde yapılan histolojik çalışma primer splenik anjiosarkom tanısını doğruladı. Hasta kemoterapi aldı fakat splenektomiden 1 yıl sonra metastazdan öldü.

## INTRODUCTION

Bone marrow is one of the common sites to be involved with solid tumors that metastasize via the bloodstream. Micrometastases can be demonstrated in the bone marrow of 30%-75% of patients with common malignancies [1]. Metastases involved in cortical bones often present with bony pain, pathologic fracture, and hypercalcemia [2]. Marrow involvement appears to be a prerequisite for the development, as bony metastases occur at the sites with hematopoietic marrow [2]. Extensive infiltration of the bone marrow may compromise hematopoietic functions. Hematologic abnormalities suggestive of marrow infiltration are peripheral cytopenia and leukoerythroblastic changes, and their occurrence is largely due to marrow replacement by tumor infiltration and reactive marrow fibrosis [2].

Bone marrow aspirate and biopsy can be used to easily diagnose medullary metastases. The incidence of bone marrow involvement varies with types of primary tumors. The solid tumors most frequently detected in bone marrow in adults are carcinomas of the breast, prostate, lungs, and gastrointestinal tract [3,4]. Angiosarcomas are rare, comprising only about 2% of all soft tissue sarcomas. Primary angiosarcomas can occur at any site of the body. The most common sites are the skin and superficial soft tissues, followed by the breast, liver, spleen, and bone. Primary splenic angiosarcoma is an uncommon primary tumor. Bone marrow metastasis in splenic angiosarcoma, however, is exceedingly rare.

We report here the pathologic findings of a patient with bone marrow metastasis of a primary unsuspected splenic angiosarcoma.

## CASE PRESENTATION

In December 2010, a 25-year-old woman complained of anemic syndrome. She presented with serious normocytic normochromic anemia with nodular splenomegaly. Bone marrow biopsy was performed. Histopathological analysis found regular bone trabeculae and delimiting medullary compartments with normal cellular richness, where the 3 myeloid lineages were represented at different maturation stages. There was a spindle proliferation delineating vascular-like slits, which were sometimes empty or contained erythrocytes. The cells presented moderate nuclear atypia, often with a prominent acidophilic nucleolus.

**Figure 1:**
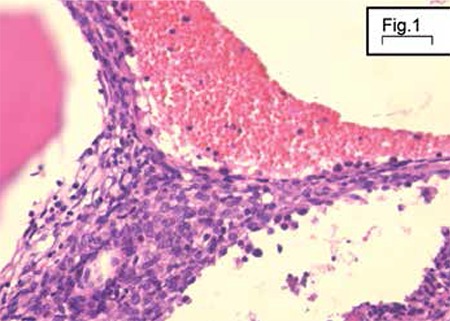
Bone marrow biopsy: “bloody” appearance with spindle cells proliferation, some cytologic atypia, and mitotic activity.

**Figure 2:**
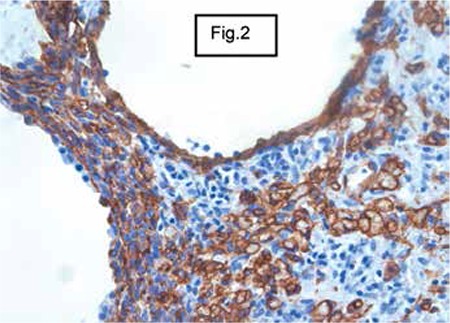
Immunohistochemestry: CD34 (+), CD31 (-), HHV8 (-).

The immunohistochemical study showed that spindle cells expressed CD34 and did not express CD31. Results for CD117 (c-kit) were difficult to interpret (Figures 1 and 2).

The medullary location of a vascular-like spindle proliferation was the most evident clue for diagnosis. Another bone marrow biopsy was recommended to better pinpoint the diagnosis. Informed consent was obtained.

In January 2011, a second bone marrow biopsy was performed. Microscopic study highlighted, in the medullary compartments, a spindle proliferation with marked vascular differentiation. This was represented by slits or sometimes large virtual vascular cavities, surrounded and partitioned by turgescent endothelial cells. Islets of residual hematopoiesis were identified; they consisted of elements belonging to the 3 myeloid lineages.

The new immunohistochemical study showed the same profile. The spindle cells did not express c-kit (CD117), with a positive internal control.

**Figure 3:**
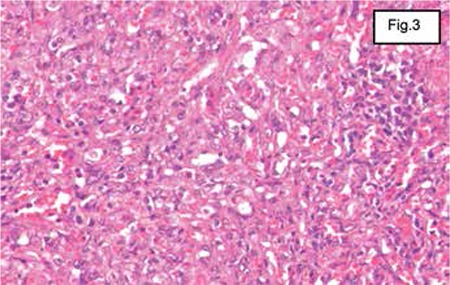
Microscopy: spindle proliferation with marked vascular differentiation. Nuclear atypia, with very few atypical cells and some calcifications.

The diagnosis of medullary location of vascular kaposiform proliferation was confirmed. Further immunostaining with HHV8 was performed and was negative (Figure 3). HIV serology and splenectomy were discussed.

An abdominal computed tomography scan showed an enlarged spleen with heterogeneous lesions. Splenectomy was done in February 2011. The spleen weighed 706 g and measured 21x14x8 cm. The outer surface was smooth. When cut, multiple whitish nodules measuring between 0.4 and 1 cm in diameter were seen. The splenic parenchyma was the site of saffron-colored deposits. At the hilum, 15 lymph nodes were isolated, measuring between 4 and 12 mm in diameter. Histological examination showed that the nodules described above corresponded to a spindle proliferation with marked vascular differentiation. Cells were sometimes epithelioid-like, presenting moderate to marked nuclear atypia, with very few atypical cells and budding nuclei, estimated at 10 mitoses per 10 fields at high magnification. Vascular slits were engorged by erythrocytes

Moreover, there were calcifications with giant cells and a small focus of necrosis.

Fourteen lymph nodes among the 15 examined were metastatic.

The final diagnosis was that of a well-differentiated angiosarcoma (grade II) of the spleen with lymph node metastases (14 N+ of 15).

The patient received a chemotherapy course. Unfortunately, the patient died 1 year after the splenectomy from metastasis.

## DISCUSSION AND REVIEW OF THE LITERATURE

Primary splenic angiosarcoma is the most common malignant non-hematopoietic tumor of the spleen [5]. These tumors are rare, highly aggressive, and lethal. Metastases tend to occur early and spread widely [6]. Metastatic diseases were found in 69% of the patients in a reported series [7]. Early metastasis from splenic angiosarcoma largely contributes to its poor prognosis. Up to 86% of patients have distant metastases at the time of presentation. Bone marrow metastasis, however, is exceptionally rare. Interestingly, Wang et al. suggested that angiosarcomas arising in the spleen have a unique propensity for bone marrow metastasis, but this is unfortunately not well documented in the literature [8]. We have found 3 cases in the literature in which primary splenic angiosarcoma was extended to the bone marrow.

The KI-67 proliferation index determines prognosis. Because splenic angiosarcoma is a rare tumor, no specific regimen of chemotherapy has been employed in enough cases to enable the drawing of a conclusion as to effects on survival [6].

Despite best efforts, the prognosis for this diagnosis is poor, with mean survival ranging from 10.3 to 14.4 months [9].

## CONCLUSION

We believe that the true incidence of bone marrow metastasis is underreported. Bone marrow aspiration and biopsy should be systematically performed in angiosarcoma patients, especially when hematological abnormalities are found on blood count.

**Conflict of Interest Statement**

The authors of this paper have no conflicts of interest, including specific financial interests, relationships, and/or affiliations relevant to the subject matter or materials included.
